# Association of tumor‐infiltrating lymphocytes before and after neoadjuvant chemotherapy with pathological complete response and prognosis in patients with breast cancer

**DOI:** 10.1002/cam4.4302

**Published:** 2021-09-25

**Authors:** Jin Hong, Weiwei Rui, Xiaochun Fei, Xiaosong Chen, Kunwei Shen

**Affiliations:** ^1^ Department of General Surgery Comprehensive Breast Health Center Ruijin Hospital Shanghai Jiao Tong University School of Medicine Shanghai China; ^2^ Chinese Academy of Sciences Key Laboratory of Tissue Microenvironment and Tumor Shanghai Institute of Nutrition and Health Chinese Academy of Sciences Shanghai China; ^3^ Department of Pathology Ruijin Hospital Shanghai Jiao Tong University School of Medicine Shanghai China

**Keywords:** breast cancer, neoadjuvant chemotherapy, pathological complete response, prognosis, tumor‐infiltrating lymphocytes

## Abstract

**Purpose:**

To evaluate the predictive and prognostic value of tumor‐infiltrating lymphocytes (TILs) before and after neoadjuvant chemotherapy (NAC) in patients with breast cancer.

**Patients and methods:**

Consecutive breast cancer patients treated with NAC between August 2008 and November 2019 were retrospectively analyzed. TIL levels were evaluated of invasive tumor samples, and high expression was defined as TILs >10%. Total pathological complete response (pCR) was defined as no invasive tumor in the breast or lymph nodes. Univariate and multivariate analyses were used to assess factors associated with pCR rate, disease‐free survival (DFS), and overall survival.

**Results:**

A total of 461 patients were included. The mean pre‐NAC TIL level was higher among patients with pCR than among patients without pCR (24.28% ± 2.34% vs. 11.34% ± 0.60%, respectively, *p* < 0.0001). The multivariate analysis demonstrated that a high pre‐NAC TIL level was an independent risk factor for a higher pCR (odds ratio = 3.92, 95% CI = 2.23–6.90, *p* < 0.001). Patients with high pre‐NAC TIL levels had a better 5‐year DFS than those with low pre‐NAC TIL levels (84.5% vs. 68.9%, HR = 0.50, 95% CI = 0.31–0.81, *p* = 0.005). The multivariate analysis showed that pre‐NAC TIL (HR = 0.48; 95% CI = 0.29–0.81, *p* = 0.006) but not post‐NAC TIL (HR = 0.89, 95% CI = 0.50–1.59, *p* = 0.699) was significantly associated with DFS among patients without pCR. Furthermore, patients with low pre‐ and post‐NAC TIL levels had a worse 5‐year DFS than those with high pre‐NAC TIL levels (HR = 2.09, 95% CI = 1.23–3.56, *p* = 0.007).

**Conclusions:**

Pre‐NAC TIL level can predict pCR and DFS in patients with breast cancer receiving NAC. For patients without pCR, pre‐NAC TIL, and TIL category change, but not post‐NAC TIL, were significantly associated with DFS.

## 1 INTRODUCTION

Over the past few decades, the immune mechanisms underlying tumor elimination and escape have been extensively studied.^1^ Immune cells infiltrating the tumor microenvironment are significantly correlated with survival in breast cancer patients.^1^ The level of tumor‐infiltrating lymphocytes (TILs) is a foremost immunobiological marker and can be classified as stromal or intra‐tumoral.^2^ The presence of abundant TILs is significantly correlated with superior survival in patients with human epidermal growth factor receptor 2 (HER2)–enriched or triple‐negative breast cancer (TNBC), and studies have revealed that the stromal TIL level is a better biomarker than the level of intra‐tumoral TIL.^2^, ^3^

Neoadjuvant chemotherapy (NAC) has been increasingly used in breast cancer patients for tumor downstaging and more breast conservation feasibility as well as to provide a window for preoperative tumor shrink, thus guiding adjuvant systemic therapy, especially for HER2^+^ and TNBC patients.^4^ Patient pathological response to NAC is significantly associated with prognosis, and those who achieved pathological complete response (pCR) had better disease‐free survival (DFS) and overall survival (OS) rates, particularly in case of aggressive tumors.^5^, ^6^, ^7^

A meta‐analysis and pooled analysis reported that increased pre‐NAC TIL concentration predicted pathological response in all of the molecular subtypes and was strongly correlated with survival in TNBC and HER2^+^ patients treated with NAC.^8^, ^9^ However, the predictive and prognostic values of the post‐NAC TIL level and TIL change before and after NAC have been rarely studied, and the results are controversial.^10^, ^11^, ^12^ Hamy et al. reported that poor DFS was observed in HER2^+^ patients with a high post‐NAC TIL level and that a decrease in TIL level after NAC was strongly associated with a better pathological response.^10^ On the contrary, Ochi et al. reported that TNBC patients with a low post‐NAC TIL level had only a numerically shorter recurrence‐free survival (RFS) that could not be predicted by a TIL change.^12^

Therefore, the clinical values and changes of TIL pre‐ and post‐NAC require further elucidation. This study aimed to explore the predictive and prognostic value of pre‐NAC, post‐NAC, and change in TIL before and after NAC in patients with breast cancer receiving NAC.

## 2 PATIENTS AND METHODS

### 2.1 Patients and treatment

Consecutive female breast cancer patients who received NAC at Ruijin Hospital Shanghai Jiao Tong University School of Medicine between August 2008 and November 2019 were retrieved from the Shanghai Jiao Tong University Breast Cancer Database and retrospectively analyzed.

All patients included in our study received at least two cycles of NAC. The NAC regimens for these patients were classified into three categories: anthracycline‐containing, taxane‐containing, and anthracycline/taxane combinations. Anti‐HER2‐targeted therapy, such as trastuzumab or trastuzumab plus pertuzumab, is recommended for patients with HER2^+^ tumors. After NAC, radical standard breast cancer surgery was performed in all patients.

### 2.2 Clinical evaluation

The clinical data of all patients were derived from the Shanghai Jiao Tong University database. The clinical tumor stages and nodal status before NAC were determined through physical examination (PE) and ultrasonography. Clinical node‐negative (cN0) was defined as no abnormal lymph nodes on ultrasound or PE or confirmed negative cytological results by fine‐needle aspiration. The American Joint Committee on Cancer staging manual (2017) was used for clinical tumor, node, metastasis staging in this study.^13^

### 2.3 Pathological assessment

Before NAC, a core needle biopsy (CNB) of the primary breast tumor was performed for diagnostic confirmation in all patients. The tumors' histopathological and immunohistochemical (IHC) characteristics were evaluated by two independent pathologists. Estrogen receptor (ER) or progesterone receptor (PR) positivity was defined if at least 1% of the invasive tumor cells stained positive on IHC. HER2 positivity was defined as CerbB‐2 3+ measured by IHC and/or HER2 amplification detected by fluorescence in situ hybridization (FISH). Breast cancer tumors were classified into three molecular subtypes based on the IHC and FISH results: hormone receptor (HR)^+^/HER2^−^, HER2^+^, and TNBC.

After NAC, the pathological response of the primary breast tumor and axillary lymph nodes was assessed of the surgical specimens. The absence of residual invasive carcinoma in the breast and lymph nodes (ypT0/isypN0) was considered total pCR. No invasive breast cancer in the breast (ypT0/is) was defined as breast pCR regardless of node status. The Miller and Payne (MP) grading system (grades 1–5) was used to grade the pathological response of the primary breast tumor after NAC.^14^

### 2.4 Stromal TILs evaluation

Considering the recommendations by the International TILs Working Group and its update, hematoxylin and eosin–stained histological slides of tumor tissues were used for the stromal TIL evaluations.^15^, ^16^ The CNB samples before NAC and surgical samples after NAC were assessed and TILs were evaluated in samples with invasive tumors. In samples without invasive tumors after NAC (breast non‐pCR), stromal TILs could not be evaluated. The stromal TIL level is reported as the percentage of the area occupied by mononuclear inflammatory cells over the total intra‐tumoral stroma area.

### 2.5 Statistical analysis

The stromal TIL levels are presented as mean ± SE. The optimal cut‐off point of the stromal TIL for total pCR prediction was determined by receiver operating characteristic (ROC) curve analysis. The Mann–Whitney *U* test was used to compare continuous variables. Two‐sided Pearson chi‐square tests were used to compare categorical variables. To determine the independent predictive factors for pCR, a multivariate logistic regression model was used.

The Kaplan–Meier method was used to analyze DFS and OS, and the log‐rank test was used for comparisons. DFS was defined as the survival period without newly diagnosed contralateral breast cancer, any local or regional recurrence, distant metastasis, secondary malignancy, or death of any cause. OS was defined as the time from the first surgery to death of any cause. To evaluate the independent factors for survival, Cox proportional hazards models were used for the multivariate analysis.

IBM SPSS statistics software version 23 (SPSS, Inc.) was used for the data assessment and statistical analysis. Images were produced using GraphPad Prism version 7.0 (GraphPad Software). Two‐sided *p* < 0.05 were considered statistically significant.

## 3 RESULTS

### 3.1 Patient characteristics

From our institution's database, the data of a total of 461 breast cancer patients for whom paired tumor tissue samples were taken before and after NAC and who were treated between August 2008 and November 2019 were retrospectively included in our study. The median patient age was 50 years (range, 21–82 years). In terms of molecular subtypes, 196 were HR^+^HER2^−^, 165 were HER2^+^, and 100 were TNBC (Table 1). The distribution of clinical stage was as follows: cT1 in 51 (11.0%), cT2 in 299 (64.9%), and cT3 or cT4 in 111 (24.1%); and cN0 in 98 (23.6%), cN1 in 213 (46.2%), and cN2 or cN3 in 150 (32.5%).

**TABLE 1:** Baseline patients' characteristics

Characteristics	Total	Pre‐NAC TILs		*p*‐value
		≤10%	>10%	
Age				0.798
<50	220	144 (48.2)	76 (46.9)	
≥50	241	155 (51.8)	86 (53.1)	
Menopausal status				0.782
Premenopausal	235	151 (50.5)	84 (51.9)	
Postmenopausal	226	148 (49.5)	78 (48.1)	
cT				0.003
1	51	25 (8.3)	26 (16.0)	
2	299	189 (63.2)	110 (67.9)	
3	78	57 (19.1)	21 (13.0)	
4	33	28 (9.4)	5 (3.1)	
cN				0.023
0	98	73 (24.4)	25 (15.4)	
1	213	131 (43.8)	82 (50.6)	
2	93	53 (17.7)	40 (24.7)	
3	57	42 (14.1)	15 (9.3)	
Pathology				0.103
IDC	371	234 (78.3)	137 (84.6)	
Others	90	65 (21.7)	25 (15.4)	
Grade				<0.001
I–II	112	85 (28.4)	27 (16.7)	
III	182	95 (31.8)	87 (53.7)	
NA	167	119 (39.8)	48 (29.6)	
ER				<0.001
Negative	204	108 (36.1)	96 (59.3)	
Positive	257	191 (63.9)	66 (40.7)	
PR				<0.001
Negative	289	159 (53.2)	130 (80.2)	
Positive	172	140 (46.8)	32 (19.8)	
HER2				<0.001
Negative	296	210 (70.2)	86 (53.1)	
Positive	165	89 (29.8)	76 (46.9)	
Ki67				
<14%	89	73 (24.4)	16 (9.9)	<0.001
≥14%	372	226 (75.6)	146 (90.1)	
Molecular subtypes				<0.001
HR^+^HER2^−^	196	155 (51.8)	41 (25.3)	
HER2^+^	165	89 (29.8)	76 (46.9)	
TNBC	100	55 (18.4)	45 (27.8)	

Abbreviations: cN, clinical nodal stage; cT, clinical tumor stage; ER, estrogen receptor; HER2, human epidermal growth factor receptor 2; HR, hormonal receptor; NAC, neoadjuvant chemotherapy; PR, progesterone receptor; TILs, tumor‐infiltrating lymphocytes; TNBC, triple‐negative breast cancer.

The treatment information for these patients are summarized in Table 2. Forty‐four patients were treated with anthracycline‐containing NAC regimens, 75 with taxane‐based regimens, and 342 with anthracycline and taxane combination therapy. Of the 165 HER2^+^ patients, 118 (71.5%) were treated with anti‐HER2^−^ targeted therapy. Regarding NAC cycles, 126 patients received no more than four cycles, 181 patients received five to seven cycles, and 154 patients received eight or more cycles. After NAC, 422 patients underwent mastectomy and 39 patients received breast‐conserving surgery. An axillary lymph node dissection was performed in 451 patients, while sentinel lymph node biopsy alone was performed in 10.

**TABLE 2:** Treatment and response for patients

Characteristics	Total	Pre‐NAC TILs		*p*‐value
		≤10%	>10%	
Neoadjuvant‐chemotherapy				0.373
Anthracycline containing	44	32 (10.7)	12 (7.4)	
Taxanes containing	75	45 (15.1)	30 (18.5)	
Anthracycline + taxanes	342	222 (74.2)	120 (74.0)	
Neoadjuvant‐targeted therapy				0.019
Yes	118	66 (22.1)	52 (32.1)	
No	343	233 (77.9)	110 (67.9)	
NAC cycles				0.510
≤4	126	87 (29.1)	39 (24.1)	
5–7	181	115 (38.5)	66 (40.7)	
≥8	154	97 (32.4)	57 (35.2)	
Breast surgery				0.132
Mastectomy	422	278 (93.0)	144 (88.9)	
BCS	39	21 (7.0)	18 (11.1)	
Axillary surgery				0.745
SLNB	10	6 (2.0)	4 (2.5)	
ALND	451	293 (98.0)	158 (97.5)	
ypTis/0				<0.001
Yes	103	38 (12.7)	65 (40.1)	
No	358	261 (87.3)	97 (59.9)	
ypTis/0N0				<0.001
Yes	74	24 (8.0)	50 (30.9)	
No	387	275 (92.0)	112 (69.1)	
Miller‐Payne				<0.001
1	18	15 (5.0)	3 (1.9)	
2	198	166 (55.5)	32 (19.7)	
3	116	69 (23.1)	47 (29.0)	
4	26	11 (3.7)	15 (9.3)	
5	103	38 (12.7)	65 (40.1)	

Abbreviations: ALND, axillary lymph node dissection; BCS, breast conservative surgery; NAC, neoadjuvant chemotherapy; NAC, neoadjuvant chemotherapy; SLNB, sentinel lymph node biopsy; TILs, tumor‐infiltrating lymphocytes.

### 3.2 Pre‐NAC TIL distributions

**FIGURE 1:**
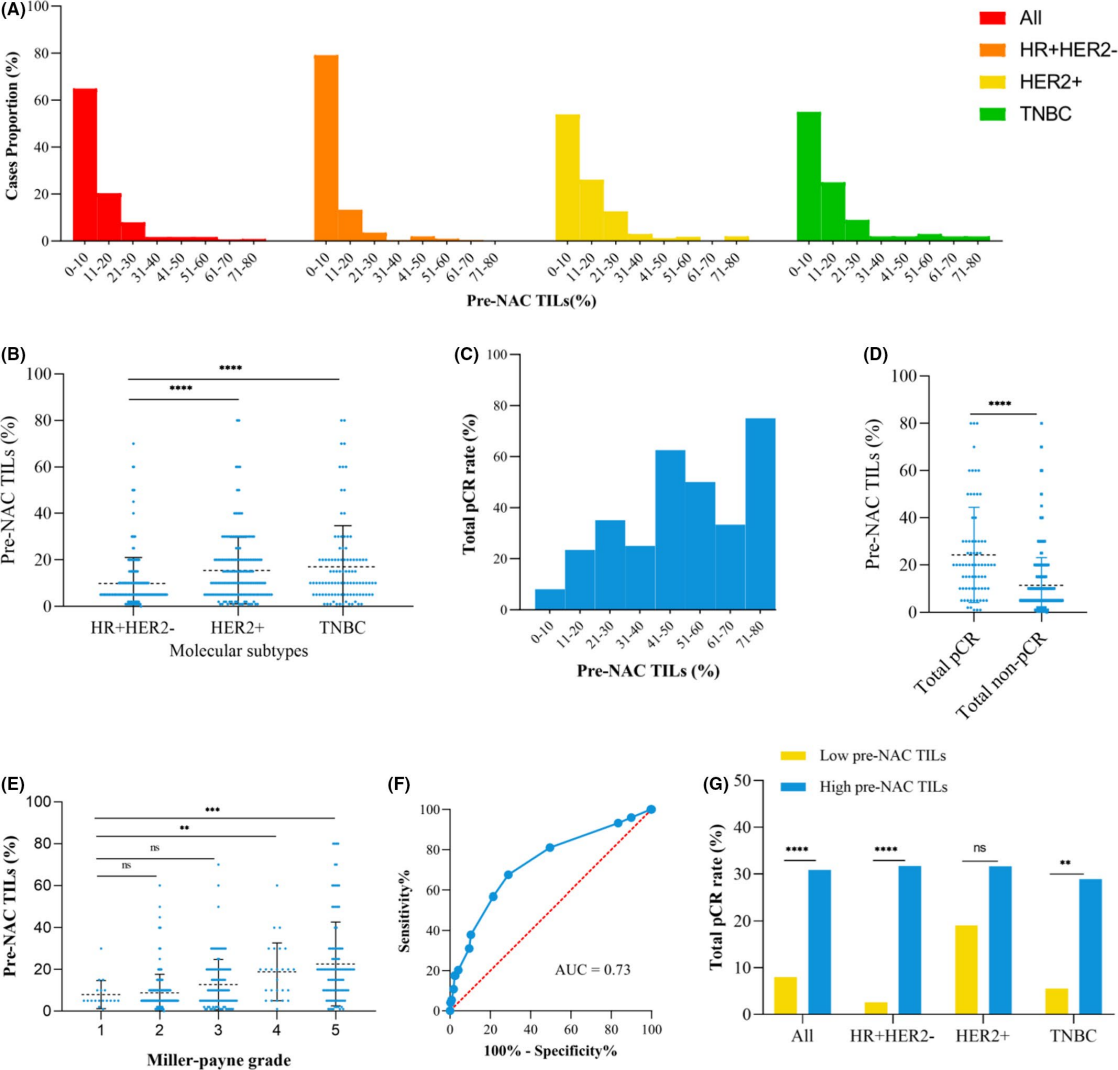
Distributions of pre‐NAC TILs and associations with pathological response. (A) Distribution of pre‐NAC TILs in all patients and the HR^+^HER2^−^, HER2^+^, and TNBC subgroups; (B) Mean pre‐NAC TIL of patients by molecular subtype; (C) Total pCR rate by 10% increment of pre‐NAC TIL; (D) Mean pre‐NAC TILs among patients with total pCR or non‐pCR; (E) Associations between pre‐NAC TIL and Miller‐Payne grade after NAC; (F) Area under the curve of pre‐NAC TIL for total pCR; (G) Total pCR rate of patients with high versus low pre‐NAC TILs. HER2, human epidermal growth factor receptor 2; HR, hormonal receptor; NAC, neoadjuvant chemotherapy; ns, nonsignificant; pCR, pathological complete response; TILs, tumor‐infiltrating lymphocytes; TNBC, triple‐negative breast cancer. *p < 0.05; **p < 0.01; ***p < 0.001; ****p < 0.0001

The mean pre‐NAC TIL level was 13.42% ± 0.66% in the whole population. The distributions of pre‐NAC TILs by 10% increments are shown in Figure 1A. Pre‐NAC TIL levels of 0%–10% were found in 299 (64.9%) tumors, 94 (20.4%) were 11%–20%, 37 (8.0%) were 21%–30%, and 31 (6.7%) patients had tumors with TILs >30%. The mean pre‐NAC TIL level was 9.86% ± 0.80% in patients with HR^+^HER2^−^ tumors, significantly lower than that of patients with HER2^+^ tumors (15.44% ± 1.11%, *p* < 0.0001) or TNBC (17.06% ± 1.77%, *p* < 0.0001) (Figure 1B; Table S1).

The total pCR rate increased as pre‐NAC TIL levels increased, as did the breast pCR rate (Figure 1C; Table S2). Regarding the association between the TIL level and total pCR rate, the optimal TIL cut‐off value was 10% on the ROC curve analysis, with an area under the curve of 0.73 (95% CI = 0.66–0.80, sensitivity = 67.6%, specificity = 71.1%, *p* < 0.0001) (Figure 1F). Compared with patients in the low pre‐NAC TIL (≤10%) subgroup, more patients in the high pre‐NAC TIL (>10%) subgroup had grade III (53.7% vs. 31.8%, *p* < 0.001), ER^−^ (59.3% vs. 36.1% *p* < 0.001), PR^−^ (80.2% vs. 53.2% *p* < 0.001), and HER2^+^ (46.9% vs. 29.8%, *p* < 0.001) tumors as well as a Ki67 level ≥14% (90.1% vs. 75.6%, *p* < 0.001) (Table 1). The proportions of HER2^+^ and TNBC tumors were higher in the high pre‐NAC TIL subgroup than in the low subgroup (HER2^+^: 46.9% vs. 29.8%, *p* < 0.001; TNBC: 27.8% vs. 18.4%, *p* < 0.001).

### 3.3 Pre‐NAC TIL and pathological response

The mean pre‐NAC TIL level was 24.4% ± 2.34% in total pCR patients and 11.34% ± 0.60% in patients without total pCR (*p* < 0.0001, Figure 1D). Compared to patients with low pre‐NAC TIL levels, patients in the high pre‐NAC TIL group had a significantly higher total pCR (ypTis/0N0) rate (30.9% vs. 8.0%, *p* < 0.001) in the whole population except for the HER2^+^ subtype (Figure 1G; Table 2). Multivariate analysis showed that ER expression, PR expression, molecular subtypes, and pre‐NAC TIL levels were significantly associated with total pCR (Tables S3 and S4). High pre‐NAC TIL level was an independent predictor of a higher total pCR rate (OR = 3.92, 95% CI = 2.23–6.90, *p* < 0.001).

Regarding the pathological response in the breast by MP grade, the mean pre‐NAC TIL was significantly higher in patients with an MP5 (22.2% ± 1.98%, *p* = 0.0001) or MP4 (18.88% ± 2.71%, *p* = 0.0013) than in patients with an MP1 (7.94% ± 1.59%) (Figure 1E). Patients in the high pre‐NAC TIL subgroup achieved a higher proportion of MP5 (40.1% vs. 12.7%), MP4 (9.3% vs. 3.7%), and MP3 (29.0% vs. 23.1%) status and a lower proportion of MP2 (19.7% vs. 55.5%) or MP1 (1.9% vs. 5.0%) than patients with a low pre‐NAC TIL level (Table 2).

### 3.4 Pre‐NAC TIL and survival

**FIGURE 2:**
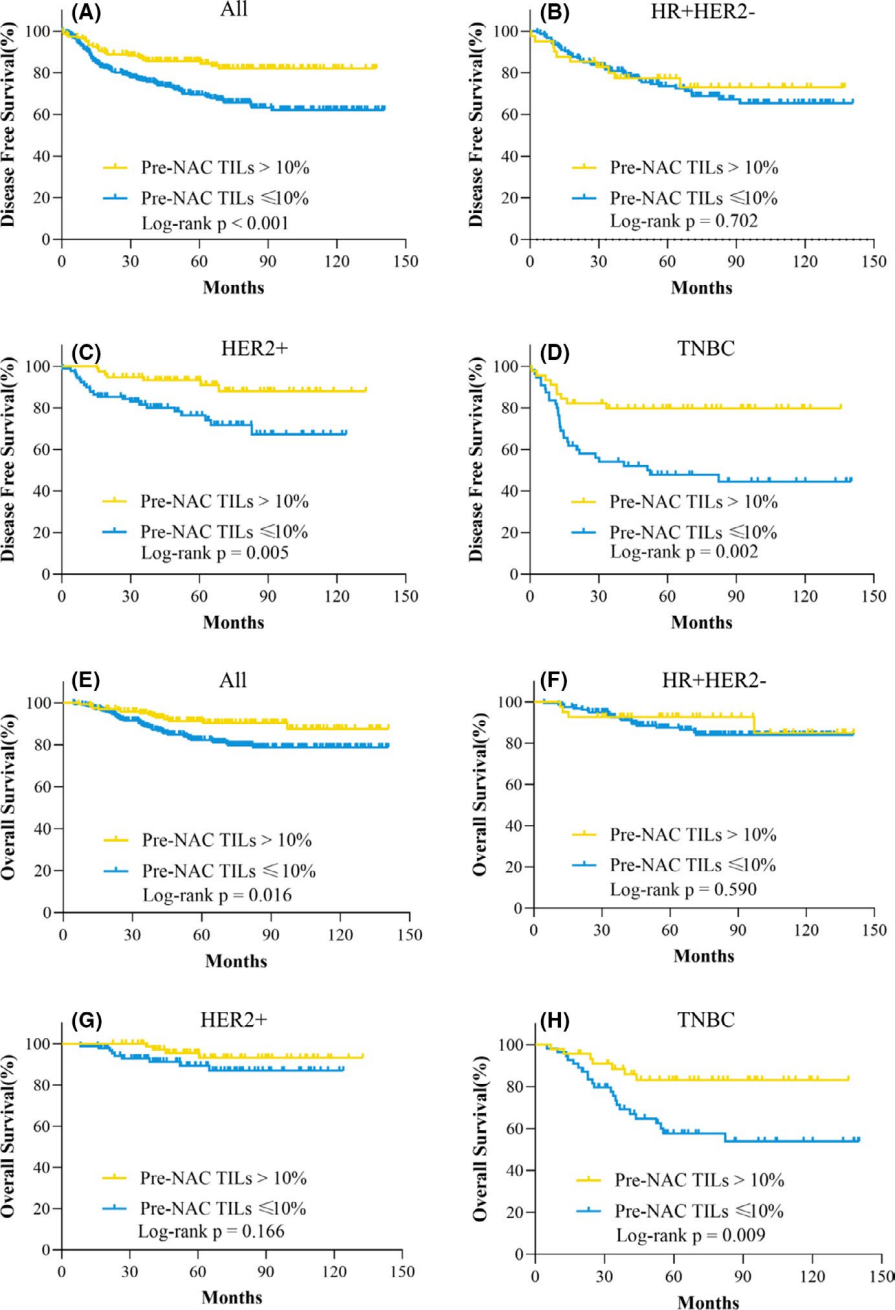
Disease‐free survival and overall survival by pre‐NAC TIL category in the whole population. (A) DFS of all patients; (B) DFS of patients with the HR^+^HER2^−^ subtype; (C) DFS of patients with the HER2^+^ subtype; (D) DFS of patients with the TNBC subtype; (E) OS of all patients; (F) OS of patients with the HR^+^HER2^−^ subtype; (G) OS of patients with the HER2^+^ subtype; (H) OS of patients with the TNBC subtype. HER2, human epidermal growth factor receptor 2; HR, hormonal receptor; NAC, neoadjuvant chemotherapy; TILs, tumor‐infiltrating lymphocytes; TNBC, triple‐negative breast cancer

The 5‐year DFS rates were 85.5% and 69.5% for patients with high and low pre‐NAC TIL levels (*p* < 0.001, Figure 2A) after a median follow‐up of 60.7 months. The multivariate analysis revealed that pre‐NAC TIL level was an independent predictor of DFS (OR = 0.50, 95% CI = 0.17–0.73, *p* = 0.005) (Tables S5 and S6). Regarding patients with different molecular subtypes, a high pre‐NAC TIL level was associated with a superior DFS compared to a pre‐NAC low TIL in the HER2^+^ group (93.3% vs. 76.3%, *p* = 0.005, Figure 2C) and the TNBC group (79.7% vs. 47.8%, *p* = 0.002, Figure 2D). Nevertheless, there was no significant difference in DFS between patients with high or low pre‐NAC TIL levels (77.3% vs. 73.5%, *p* = 0.702, Figure 2B) in the HR^+^HER2^−^ group.

The 5‐year OS rate was 91.3% among patients with a high pre‐NAC TIL, significantly higher than that in patients with a low pre‐NAC TIL (82.3%, *p* = 0.016, Figure 2E). Nevertheless, the subgroup analysis revealed significant differences in OS only in the TNBC subgroup (83.1% vs. 57.5%, *p* = 0.009, Figure 2H). The multivariate analysis showed that the pre‐NAC TIL level was only marginally associated with OS (OR = 0.54, 95% CI = 0.29–1.01, *p* = 0.053) (Tables S7 and S8).

### 3.5 Post‐NAC TIL, pathological response, and survival

**FIGURE 3:**
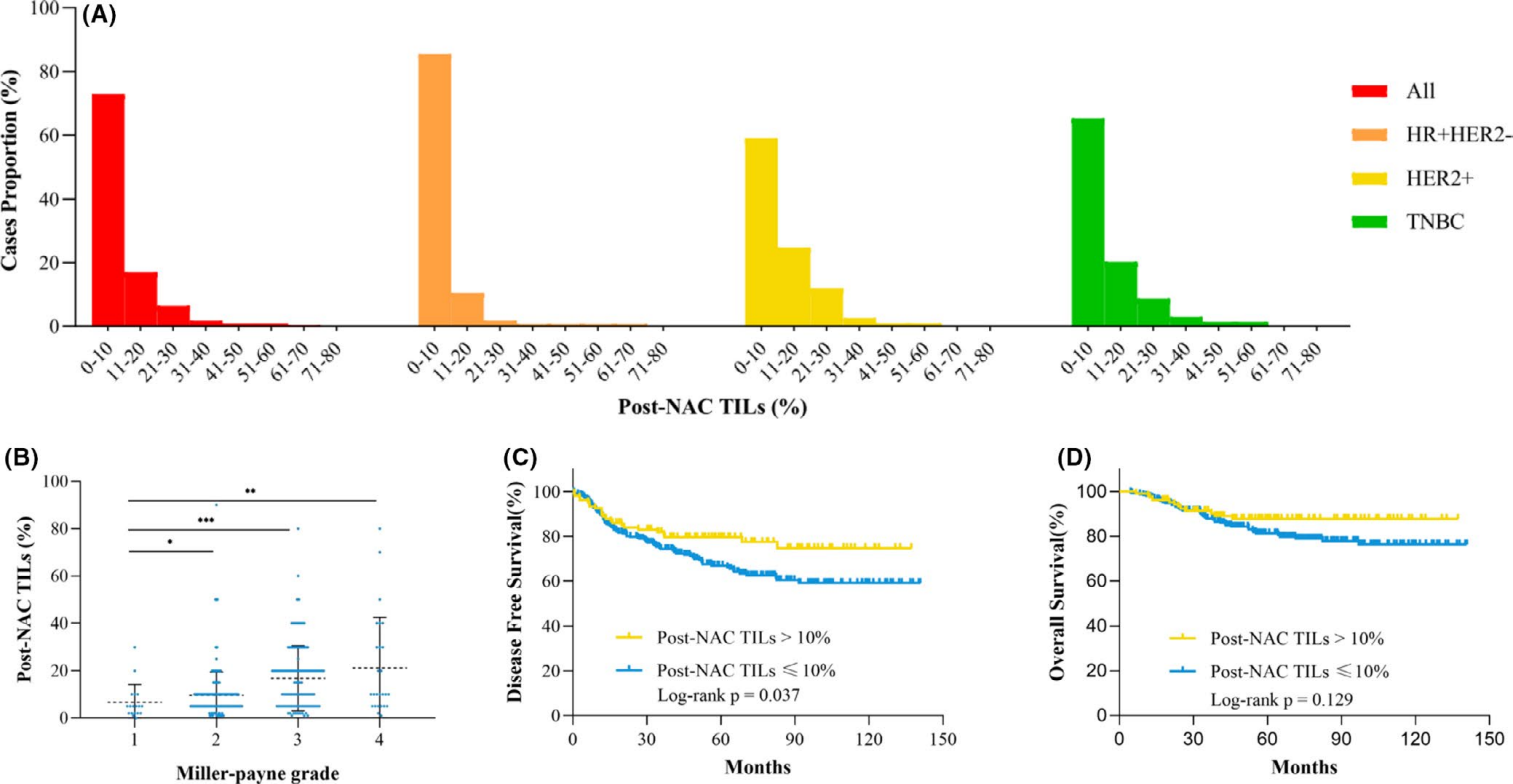
Distributions of post‐NAC TILs and associations with pathological response and survival among patients with invasive residual breast tumor after NAC. (A) Distribution of post‐NAC TILs in all patients and the HR^+^HER2^−^, HER2^+^, and TNBC subgroups; (B) associations between post‐NAC TIL and Miller‐Payne grade after NAC; (C) disease‐free survival by post‐NAC TIL category; (D) overall survival by post‐NAC TIL category. HER2, human epidermal growth factor receptor 2; HR, hormonal receptor; NAC, neoadjuvant chemotherapy; TILs, tumor‐infiltrating lymphocytes; TNBC, triple‐negative breast cancer

A total of 103 patients who achieved breast pCR and the remaining 358 patients without pCR were included in the post‐NAC TIL analysis. The distribution of post‐NAC TILs was as follows: 0%–10% in 261 (72.9%) tumors, 11%–20% in 61 (17.0%) tumors, 21%–30% in 23 (6.4%) tumors, and >30% in 13(3.6%) tumors (Figure 3A). The proportion of post‐NAC TILs >10% was higher in HER2^+^ and TNBC tumors than in HR^+^HER2^−^ tumors. In terms of MP grade in patients with residual tumors, the mean post‐NAC TIL level was significantly higher in tumors of MP4 (21.27% ± 4.15%, *p* = 0.0015), MP3 (16.86% ± 1.28%, *p* = 0.0002), and MP2 (9.66% ± 0.70%, *p* = 0.02) grades than those of MP1 (6.72% ± 1.77%) grade (Figure 3B).

Patients with high post‐NAC TILs had a better DFS than those with low post‐NAT TILs (79.5% vs. 66.9%, *p* = 0.037, Figure 3C). However, the multivariate analysis (Model 1) showed that post‐NAC TIL level was not an independent predictive factor for DFS (OR = 0.89, 95% CI = 0.50–1.59, *p* = 0.688) (Table 3). However, pre‐NAC‐TIL level was still significantly associated with DFS in breast non‐pCR patients (OR = 0.48, 95% CI = 0.29–0.81, *p* = 0.006). There was no significant difference in OS between patients with high post‐NAC TIL levels and those with low post‐NAC TIL levels (87.8% vs. 81.2%, *p* = 0.129) (Figure 3D).

**TABLE 3:** Multivariate analysis of factors associated with DFS in breast non‐pCR patients

Characteristics	Multivariate (Model 1^a^)			Multivariate (Model 2^b^)		
	HR	95% CI	*p*‐value	HR	95% CI	*p*‐value
cT			0.006			0.006
1	1			1		
2	0.98	0.44–2.18	0.962	0.97	0.44–2.17	0.945
3	1.68	0.71–3.97	0.238	1.66	0.70–3.96	0.250
4	2.53	1.01–6.34	0.047	2.51	0.99–6.33	0.051
cN			0.007			0.007
0	1			1		
1	0.93	0.56–1.54	0.782	0.93	0.56–1.54	0.784
2	1.41	0.76–2.59	0.274	1.41	0.76–2.59	0.274
3	2.35	1.27–4.34	0.006	2.35	1.27–4.35	0.006
Grade			0.001			0.001
I–II	1			1		
III	2.88	1.65–5.01	<0.001	2.85	1.62–5.01	<0.001
NA	1.95	1.11–3.43	0.021	1.93	1.09–3.43	0.021
ER						
Negative	1			1		
Positive	0.91	0.43–1.95	0.815	0.91	0.43–1.94	0.805
HER2						
Negative	1			1		
Positive	1.36	0.71–2.60	0.356	1.44	0.74–2.82	0.280
Molecular subtypes			<0.001			<0.001
ER^+^HER2^−^	1			1		
HER2^+^	1.43	0.75–2.73	0.284	1.44	0.74–2.82	0.280
TNBC	2.67	1.68–4.27	<0.001	2.69	1.68–4.30	<0.001
Pre‐NAC TILs						
≤10%	1					
>10%	0.48	0.29–0.81	0.006			
Post‐NAC TILs						
≤10%	1					
>10%	0.89	0.50–1.59	0.688			
TILs changes						0.023
Group A				1		
Group B				1.97	0.89–4.34	0.094
Group C				2.09	1.23–3.56	0.007
Neoadjuvant‐targeted therapy						
No	1			1		
Yes	0.38	0.17–0.84	0.016	0.38	0.17–0.84	0.017
NAC cycles			0.185			0.182
≤4	1			1		
5–7	1.04	0.65–1.66	0.871	1.03	0.65–1.65	0.890
≥8	0.64	0.36–1.15	0.135	0.64	0.35–1.14	0.130

Abbreviations: cN, clinical nodal stage; cT, clinical tumor stage; ER, estrogen receptor; HER2, human epidermal growth factor receptor 2; HR, hormonal receptor; NAC, neoadjuvant chemotherapy; TILs, tumor‐infiltrating lymphocytes; TNBC, triple negative breast cancer.

^a^ Model 1: the Cox proportional hazards model comprise pre‐NAC TILs, post‐NAC TILs, and other clinicopathological factors.

^b^ Model 2: the Cox proportional hazards model comprise changes of TILs and other clinicopathological factors. Group A: pre‐NAC TILs > 10% regardless of post‐NAC TILs; Group B: pre‐NAC TILs ≤ 10% and post‐NAC TILs > 10%; Group C: pre‐NAC and post‐NAC TILs ≤ 10%.

### 3.6 TIL level changes, pathological response, and survival

**FIGURE 4:**
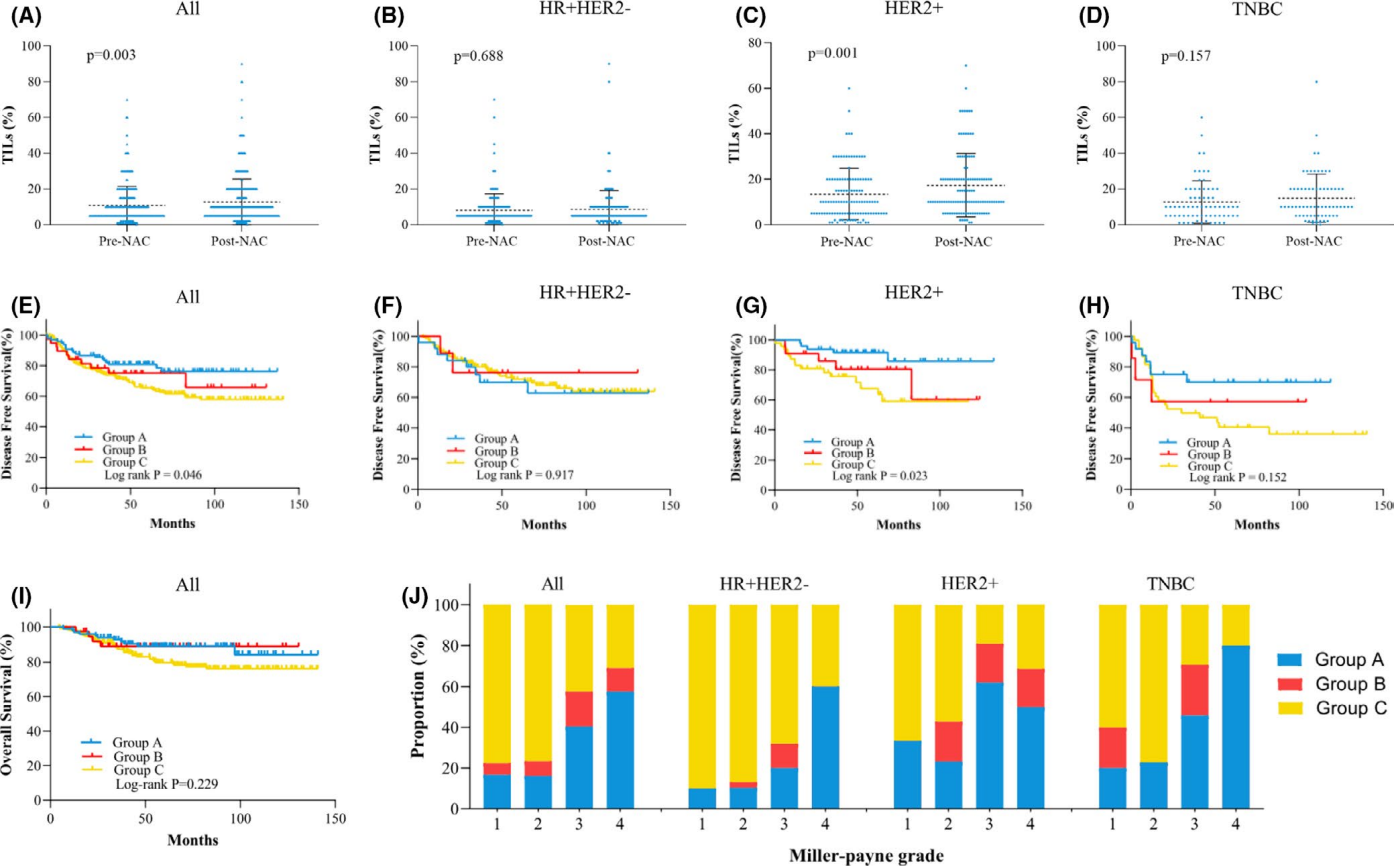
Changes in TILs before versus after NAC and associations with survival in patients with invasive residual breast tumors after NAC. (A) Changes in TILs in all patients; (B) changes in TILs in the HR^+^HER2^−^ subgroup; (C) changes in TILs in the HER2^+^ subgroup; (D) changes in TILs in the TNBC subgroup; (E) DFS by change in TIL category in all patients; (F) DFS by change in TIL category in the HR^+^HER2^−^ subgroup; (G) DFS by change in TILs category in the HER2^+^ subgroup; (H) DFS by change in TIL category in the TNBC subgroup; (I) OS by change in TILs in all patients; (J) distributions of changes in TILs in all patients by molecular subgroup. DFS, disease‐free survival; HER2, human epidermal growth factor receptor 2; HR, hormonal receptor; OS, overall survival; TILs, tumor‐infiltrating lymphocytes; TNBC, triple‐negative breast cancer; NAC, neoadjuvant chemotherapy. *Group A: pre‐NAC TILs > 10% regardless of post‐NAC TILs; Group B: pre‐NAC TILs ≤ 10% and post‐NAC TILs > 10%; Group C: pre‐NAC and post‐NAC TILs ≤ 10%

In patients with residual disease in the breast, the mean post‐NAC TIL level was significantly higher than the mean pre‐NAC TIL level (12.69% ± 0.68% vs. 10.77% ± 0.57%, *p* = 0.003) (Figure 4A). In the subgroup analysis, a significantly elevated post‐NAC TIL level was observed only in HER2^+^ patients (17.39% ± 1.29% vs. 13.45% ± 1.05%, *p* = 0.001) (Figure 4C).

Based on the changes in TIL categories before and after NAC, patients were then categorized into three groups: group A (pre‐NAC TILs >10% regardless of post‐NAC TIL level), group B (pre‐NAC TILs ≤10%, post‐NAC TILs >10%), and group C (pre‐NAC and post‐NAC TILs ≤10%). The associations between TIL changes and pathological responses in the breast are presented in Figure 4J. The proportions of group C were lower in tumors with MP4 and MP3 grades (MP1: 77.8%; MP2: 76.8%; MP3: 42.2%; MP4: 30.8%, *p* < 0.001). Similar trends were observed in the subgroup analysis of the different molecular subtypes.

The 5‐year DFS rates were 80.8%, 75.1%, and 65.5% for patients in groups A, B, and C, respectively (*p* = 0.046, Figure 4E). Cox model 2 showed that patients in group C had significantly worse 5‐year DFS than those in group A (HR = 2.09, 95% CI = 1.23–3.56, *p* = 0.007, Table 3). A statistically significant difference in 5‐year DFS was found in patients with the HER2^+^ subtype: 91.5% in group A, 80.5% in group B, and 67.6% in group C (*p* = 0.023, Figure 4G). However, were no significant differences in 5‐year OS among the three groups: 88.9% in group A, 88.8% in group B, and 79.7% in group C (*p* = 0.229, Figure 4I).

## 4 DISCUSSION

The current study revealed that a high pre‐NAC TIL level was associated with a high pCR rate and favorable prognosis in breast cancer patients receiving NAC treatment. In patients without breast pCR, high pre‐NAC and post‐NAC TIL levels were associated with a higher MP grade. Moreover, the pre‐NAC TIL and changes in TIL category, but not post‐NAC TIL, were independently correlated with DFS in patients with residual disease.

The tumor microenvironment encompasses tumor cells and other various noncancerous cells, such as immune cells and fibroblasts.^17^, ^18^ The immune system plays a pivotal role in tumor evolution and involves multiple immune cells, such as T cells, neutrophils, natural killer cells, macrophages, and myeloid‐derived suppressor cells.^1^ TILs, the foremost mononuclear immune cells infiltrating the tumor microenvironment, and are strongly associated with breast cancer prognosis.^2^ In patients treated with NAC, the pooled analysis showed that high TIL levels could predict pathological response in all breast cancer subtypes and were associated with longer DFS in the TNBC and HER2^+^ but not the luminal‐HER2^−^ subtype.^3^, ^8^ Our study confirmed that a high pre‐NAC TIL level was significantly associated with higher pCR rates in the whole population regardless of molecular subtype. Analogously, patients with a high pre‐NAC‐TIL level had superior DFS, particularly in the HER2^+^ and TNBC subgroups but not in the HR^+^HER2^−^ subgroup. In the univariate analysis, better OS was also observed among patients with a high pre‐NAC TIL, but this was only marginally significant in the multivariate analysis.

The optimal cut‐off value for defining high stromal TIL levels varied across different studies. The concept of lymphocyte‐predominant breast cancer (LPBC), defined as tumors that contain more lymphocytes than tumor cells, has been used in multiple studies and features thresholds of 50%–60%.^15^ HER2^+^ or TNBC patients with LPBC had an increased pCR rate or survival benefit compared with non‐LPBC patients.^19^, ^20^, ^21^ However, the proportion of LPBC is relatively low, ranging from 6% in HR^+^HER2^−^ tumors to 16% in HER2^+^ tumors and 20% in TNBC.^22^ In our cohort, only seven of 461 (1.6%) tumors were identified as LPBC (pre‐NAC TILs > 60%): 1 (0.5%) in the HR^+^HER2^−^ subgroup, 2 (2.0%) in the HER2^+^ subgroup, and 4 (4.0%) in the TNBC subgroup. Therefore, an optimal cut‐off point was needed to clarify the patients in our cohort. Based on the ROC curve analysis, the optimal cut‐off value was 10% in our study, close to the median TIL level. The cut‐off values varied due to different subpopulations and study aims in different studies.^8^ Due to the relatively low tumor immune infiltrates in breast cancer, the median TIL level ranged from 5% to 14.1%.^23^, ^24^, ^25^, ^26^, ^27^ Salgado et al. set the TIL cut‐off at 5% and found that levels greater than 5% were associated with higher pCR rates in the NeoALTTO trial.^27^ Liu et al. demonstrated that a TIL threshold of 30% was an independent predictor for pCR and DFS in HER2^+^ patients treated with trastuzumab.^28^ In a pooled analysis, TILs were categorized into three groups: low (1%–10%), intermediate (11%–59%), and high (≥60%).^8^ According to the international TILs working group, the TIL level was primarily regarded as a continuous variable, therefore, a universal cut‐off might not exist. We are in favor of the classification for the TIL level as low, intermediate, and high.

Few studies have explored the predictive and prognostic value of post‐NAC TILs, and controversial results have been presented. Luen et al. found that a lower post‐NAC TIL level was significantly associated with increasing ypT and ypN scores in TNBC patients.^29^ Inversely, Hamy et al. revealed that a high post‐NAC TIL level was correlated with aggressive characteristics in HER2^+^ but not TNBC or luminal subtype tumors.^10^ As for prognostic value, some studies showed that a higher post‐NAC TIL level was associated with better RFS rates in HER2^+^ and TNBC patients.^11^, ^29^ Hamy et al. reported that post‐NAC TIL level was not associated with DFS in the whole population but was for the HER2^+^ subtype.^10^ The current study was the first to analyze the correlation between post‐NAC TIL levels and post‐NAC pathological response MP grade. A higher post‐NAC TIL level was significantly associated with a better pathological response in the breast for patients with residual diseases. This result was consistent with the predictive value of pre‐NAC TIL levels. Our study also showed that post‐NAC TIL level was associated with DFS for the entire population in the univariate analysis. However, the subgroup analysis showed no significant differences among the different molecular subtypes (Figure S1). The multivariate analysis revealed that post‐NAC TIL was not significantly associated with DFS, while the pre‐NAC TIL level was still an independent predictor of DFS in patients with residual invasive tumors. This finding implies that the tumor immune microenvironment differs after versus before NAC. TIL compositions are complex, and Lo et al. revealed that chemotherapy augmented the pre‐existing TIL response but failed to relieve major immune‐suppressive mechanisms, which might be correlated with impairment of the prognostic value of post‐NAC TIL.^30^

Changes in stromal TILs before and after NAC have not been clearly elucidated. Two retrospective studies revealed that the post‐NAC TIL level was lower than the pre‐NAC TIL level.^10^, ^31^ However, in our study, the mean post‐NAC TIL level was higher than the mean pre‐NAC TIL level, mainly among the HER2^+^ patients. Meanwhile, no significant differences were found in the HR^+^HER2^−^ and TNBC subgroups. This might be attributed to targeted therapy, as another study also showed increased stromal TIL levels after administration of ado‐trastuzumab. Therefore, an elevated TIL level after NAC may be a biomarker for anti‐HER2‐targeted therapy. Nonetheless, absolute changes in TILs were not significantly associated with DFS (Figure S2), which was similar to the conclusions made by Ochi et al.^12^ From the distributions of TIL values, the analysis by category rather than absolute change was a better method. As patients with high pre‐NAC TIL levels had a favorable prognosis, we further explored the associations between changes in TIL categories and DFS in patients with low pre‐NAC TIL levels. We found that a high post‐NAC TIL level was associated with better DFS in patients with a low pre‐NAC TIL and that patients with a low pre‐ and post‐NAC TIL had the worst prognosis. The underlying mechanism for this requires further exploration. Park et al. revealed that just one cycle of NAC induced more TILs, while residual tumors were immune suppressed at the end of treatment.^32^ Kaewkangsadan et al. found that levels of TIL and CD8^+^, CD4^+^, CTLA‐4^+^ stromal T cell, and CD8^+^/FOXP3^+^ ratios were associated with a high pCR and that NAC significantly reduced CD4^+^, FOXP3^+^, and CTLA‐4^+^ T‐cell counts.^33^ Therefore, an increase in TIL levels and CD8^+^ T‐cell counts in response to NAC may contribute to this result, especially in HER2^+^ patients.^32^

There are several limitations to our study. First, as 93.3% of patients had TILs ≤ 30% and only 6.7% had levels of 31%–80%, we analyzed the stromal TIL level as a categorical rather than continuous variable. Second, among the HER2^+^ patients, 28.5% did not receive anti‐HER2‐targeted therapy, which might have influenced our results. Third, as a retrospective study, our patients' treatment regimens and cycles were diverse and the evaluation timing for post‐NAC TIL was not unified.

## 5 CONCLUSIONS

Our study found that a high pre‐NAC TIL level was significantly associated with a high pCR rate in breast cancer patients treated with NAC regardless of molecular subtype. A high pre‐NAC TIL level was also significantly associated with better DFS in patients with HER2^+^ or TNBC. In patients without breast pCR, both high pre‐ and post‐NAC TIL levels were associated with a higher MP grade after NAC. However, only the pre‐NAC TIL level and TIL changes before and after NAC, rather than the post‐NAC TIL level, were independent prognostic factors for DFS in patients with residual disease, indicating that further TIL evaluation after NAC may guide further clinical management.

## CONFLICT OF INTEREST

All authors declare that they have no conflict of interest.

## ETHICS APPROVAL

The protocol was reviewed and approved by the independent ethical committee/institutional review board of Shanghai Ruijin Hospital affiliated with Shanghai Jiao Tong University School of Medicine.

## CONSENT FOR PARTICIPATE

All procedures performed in studies involving human participants were in accordance with the ethical standards of the committee and with the 1964 Helsinki Declaration and its later amendments or comparable ethical standards.

## Supporting information

Fig S1

Table S1‐S4

## Data Availability

The datasets used during the current study are available from the corresponding author upon reasonable request.
