# Supplementary material for: Association of tumor‐infiltrating lymphocytes before and after neoadjuvant chemotherapy with pathological complete response and prognosis in patients with breast cancer
Source: Cancer Med. 2021 Sep 25;10(22):7921–33. doi: 10.1002/cam4.4302 (PMC8607245; doi:10.1002/cam4.4302)
Supplement: Supplementary file 1 — Fig S1 [file CAM4-10-7921-s002.docx]

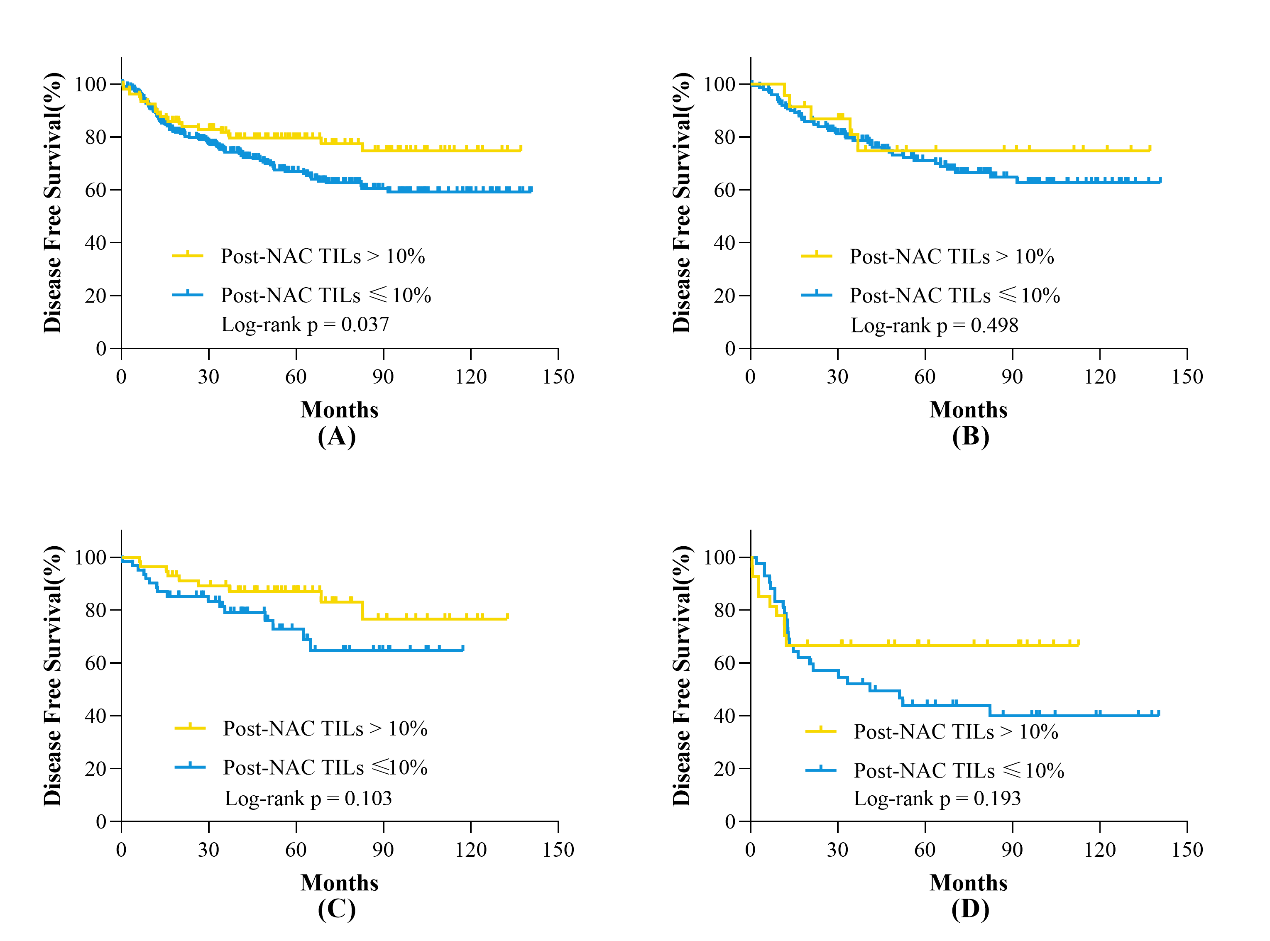


**Fig S1. Disease free survival by post-NAC TILs in patients with invasive residual breast tumor after NAC.** (A) All patients, (B) HR+HER2- subtype, (C) HER2+ subtype, (D) TNBC subtype.

Abbreviations: TILs, tumor infiltrating lymphocytes; HR, hormonal receptor; HER2, human epidermal growth factor receptor 2; TNBC, triple negative breast cancer; NAC, neoadjuvant chemotherapy.


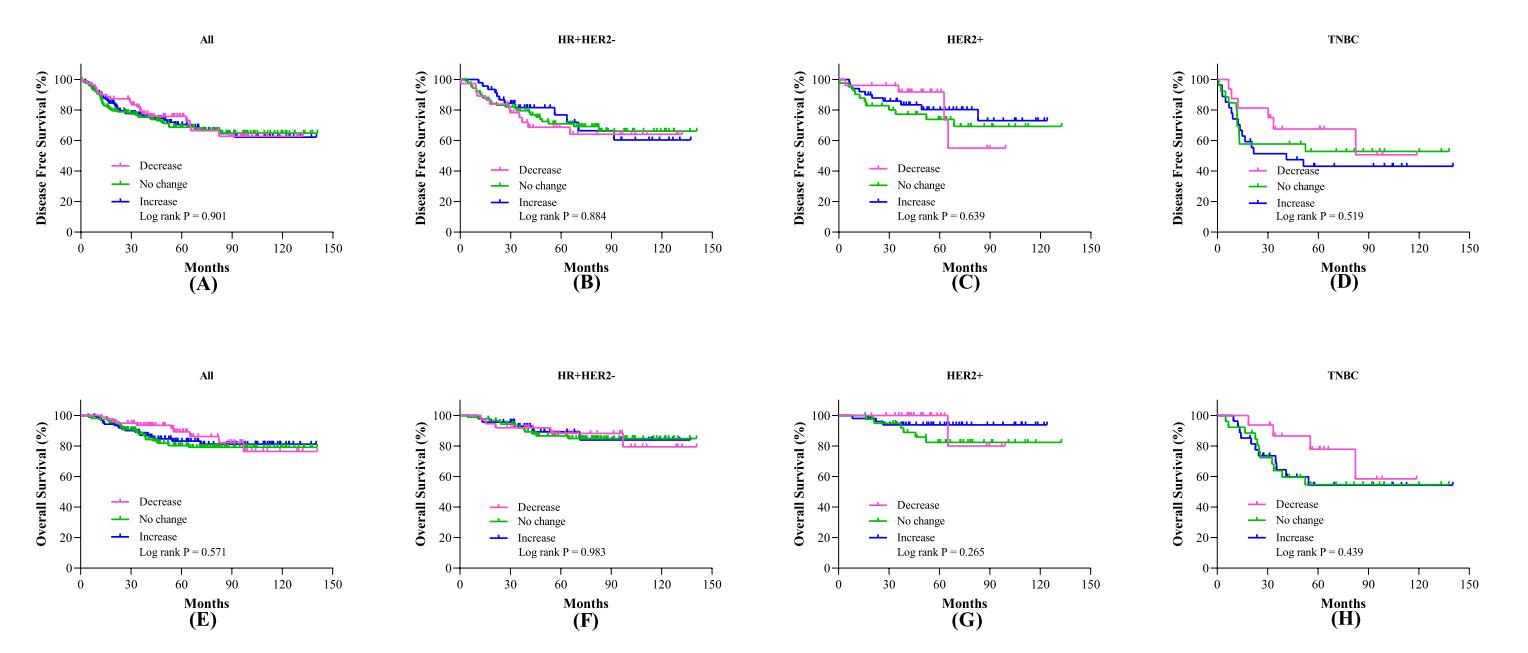


**Fig S2. Disease free survival and Overall survival by absolute changes of TILs category in breast non-pCR patients with different molecular subtypes.** (A) DFS in all patients, (B) DFS in patients with the HR+HER2- subtype, (C) DFS in patients with the HER2+ subtype, (D) DFS in patients with the TNBC subtype, (E) OS in all patients, (F) OS in patients with the HR+HER2- subtype, (G) OS in patients with the HER2+ subtype, (H) OS in patients with the TNBC subtype.

Abbreviations: TILs, tumor infiltrating lymphocytes; HR, hormonal receptor; HER2, human epidermal growth factor receptor 2; TNBC, triple negative breast cancer; NAC, neoadjuvant chemotherapy.
