# Supplementary material for: Association of tumor‐infiltrating lymphocytes before and after neoadjuvant chemotherapy with pathological complete response and prognosis in patients with breast cancer
Source: Cancer Med. 2021 Sep 25;10(22):7921–33. doi: 10.1002/cam4.4302 (PMC8607245; doi:10.1002/cam4.4302)
Supplement: Supplementary file 2 — Table S1‐S4 [file CAM4-10-7921-s001.docx]

**Table S1. Pre-NAC TILs in different molecular subtypes.**

| Pre-NAC TILs (%) | Total No. | Molecular subtypes | | |
| --- | --- | --- | --- | --- |
|  |  | HR+HER2- | HER2+ | TNBC |
| 0-10 | 299(64.9) | 155(79.1) | 89(53.9) | 55(55.0) |
| 11-20 | 94(20.4) | 26(13.3) | 43(26.1) | 25(25.0) |
| 21-30 | 37(8.0) | 7(3.6) | 21(12.7) | 9(9.0) |
| 31-40 | 8(1.7) | 1(0.5) | 5(3.0) | 2(2.0) |
| 41-50 | 8(1.7) | 4(2.0) | 2(1.2) | 2(2.0) |
| 51-60 | 8(1.7) | 2(1.0) | 3(1.8) | 3(3.0) |
| 61-70 | 3(0.7) | 1(0.5) | 0(0.0) | 2(2.0) |
| 71-80 | 4(0.9) | 0(0.0) | 2(2.0) | 2(2.0) |

Abbreviations: TILs, tumor infiltrating lymphocytes; HR, hormonal receptor; HER2, human epidermal growth factor receptor 2; TNBC, triple negative breast cancer; NAC, neoadjuvant chemotherapy.

**Table S2. The pCR rate in patients with consecutive pre-NAC TILs categories.**

| Pre-NAC TILs (%) | Patients No. | ypTis/0 (No.) | bpCR rate | ypTis/0ypN0 (No.) | tpCR rate |
| --- | --- | --- | --- | --- | --- |
| 0-10 | 299 | 38 | 12.7% | 24 | 8.0% |
| 11-20 | 94 | 33 | 35.1% | 22 | 23.4% |
| 21-30 | 37 | 14 | 37.8% | 13 | 35.1% |
| 31-40 | 8 | 2 | 25.0% | 2 | 25.0% |
| 41-50 | 8 | 5 | 62.5% | 5 | 62.5% |
| 51-60 | 8 | 5 | 62.5% | 4 | 50.0% |
| 61-70 | 3 | 2 | 66.7% | 1 | 33.3% |
| 71-80 | 4 | 4 | 100.0% | 3 | 75.0% |

Abbreviations: pCR, pathological complete response; TILs, tumor infiltrating lymphocytes; NAC, neoadjuvant chemotherapy.

**Table S3. Univariate analysis of factors associated with total pCR.**

| Characteristics | Total |
| --- | --- |
| Age | 0.177 |
| Menopausal status | 0.146 |
| cT | 0.197 |
| cN | 0.072 |
| Pathology | 0.434 |
| Grade | **0.040** |
| ER | **<0.001** |
| PR | **<0.001** |
| HER2 | **<0.001** |
| Ki67 | **0.008** |
| Molecular subtypes | **<0.001** |
| Pre-NAC TILs | **<0.001** |
| NAC regimens | **0.008** |
| Neoadjuvant-targeted therapy | **<0.001** |
| NAC cycles | 0.463 |

Abbreviations: TILs, tumor infiltrating lymphocytes; cT, clinical tumor stage; cN, clinical nodal stage; ER, estrogen receptor; PR, progesterone receptor; HER2, human epidermal growth factor receptor 2; NAC, neoadjuvant chemotherapy.

**Table S4. Multivariate analysis of factors associated with total pCR.**

| Characteristics | Multivariate | | |
| --- | --- | --- | --- |
|  | OR | 95%CI | p-value |
| ER |  |  |  |
| Negative | 1 |  |  |
| Positive | 0.39 | 0.15-1.00 | **0.049** |
| PR |  |  |  |
| Negative | 1 |  |  |
| Positive | 0.24 | 0.09-0.62 | **0.003** |
| HER2 |  |  |  |
| Negative | 1 |  |  |
| Positive | 0.68 | 0.25-1.86 | 0.450 |
| Ki67 |  |  |  |
| <14% | 1 |  |  |
| ≥14% | 1.46 | 0.57-3.72 | 0.431 |
| Grade |  |  | 0.660 |
| I-II | 1 |  |  |
| III | 1.05 | 0.50-2.22 | 0.902 |
| NA | 0.78 | 0.35-1.73 | 0.539 |
| Molecular subtypes |  |  | **0.017** |
| HR+HER2- | 1 |  |  |
| HER2+ | 0.68 | 0.25-1.86 | 0.450 |
| TNBC | 0.25 | 0.07-0.86 | **0.028** |
| Pre-NAC TILs |  |  |  |
| ≤10% | 1 |  |  |
| >10% | 3.92 | 2.23-6.90 | **<0.001** |
| Neo-chemotherapy |  |  | 0.204 |
| Anthracycline | 1 |  |  |
| Taxanes | 6.76 | 0.82-55.65 | 0.076 |
| Anthracycline+taxanes | 5.52 | 0.71-42.77 | 0.102 |
| Neo-targeted therapy |  |  |  |
| No | 1 |  |  |
| Yes | 1.16 | 0.47-3.43 | 0.646 |

Abbreviations: TILs, tumor infiltrating lymphocytes; ER, estrogen receptor; PR, progesterone receptor; HER2, human epidermal growth factor receptor 2; HR, hormonal receptor; TNBC, triple negative breast cancer.

**Table S5. Univariate analysis of factors associated with DFS in whole population and breast non-pCR patients.**

| Characteristics | Whole population | Non-pCR patients |
| --- | --- | --- |
| Age | 0.851 | 0.880 |
| Menopausal status | 0.942 | 0.958 |
| cT | **<0.001** | **0.004** |
| cN | **0.016** | **0.012** |
| pathology | 0.185 | 0.473 |
| Grade | **0.037** | **0.008** |
| ER | 0.421 | **0.043** |
| PR | 0.354 | 0.065 |
| HER2 | **0.004** | **0.023** |
| Ki67 | 0.261 | 0.955 |
| Molecular subtypes | **0.001** | **<0.001** |
| Pre-NAC TILs | **<0.001** | **0.019** |
| NAC regimens | 0.068 | 0.060 |
| Neo-targeted therapy | **<0.001** | **0.007** |
| NAC cycles | **0.009** | **0.039** |
| Breast surgery | 0.084 | 0.188 |
| Axillary surgery | 0.815 | 0.929 |
| ypTis/0N0 | **<0.001** | / |
| Post-NAC TILs | / | **0.037** |
| Changes in TILs | / | **0.046** |

Abbreviations: TILs, tumor infiltrating lymphocytes; cT, clinical tumor stage; cN, clinical nodal stage; ER, estrogen receptor; PR, progesterone receptor; HER2, human epidermal growth factor receptor 2; NAC, neoadjuvant chemotherapy.

**Table S6. Multivariate analysis of factors associated with DFS in whole population.**

| Characteristics | Multivariate | | |
| --- | --- | --- | --- |
|  | OR | 95%CI | p-value |
| cT |  |  | **<0.001** |
| 1 | 1 |  |  |
| 2 | 1.08 | 0.53-2.19 | 0.840 |
| 3 | 1.99 | 0.91-4.34 | 0.085 |
| 4 | 3.39 | 1.49-7.71 | **0.004** |
| cN |  |  | **0.022** |
| 0 | 1 |  |  |
| 1 | 0.94 | 0.59-1.52 | 0.811 |
| 2 | 1.21 | 0.68-2.16 | 0.513 |
| 3 | 2.09 | 1.17-3.74 | **0.013** |
| HER2 |  |  |  |
| Negative | 1 |  |  |
| Positive | 1.17 | 0.66-2.08 | 0.594 |
| Grade |  |  | **0.004** |
| I-II | 1 |  |  |
| III | 2.43 | 1.44-4.11 | **0.001** |
| NA | 2.01 | 1.18-3.41 | **0.010** |
| Molecular subtypes |  |  | **0.001** |
| HR+HER2- | 1 |  |  |
| HER2+ | 1.64 | 0.90-3.02 | 0.109 |
| TNBC | 2.27 | 1.47-3.52 | **<0.001** |
| Pre-NAC TILs |  |  |  |
| ≤10% | 1 |  |  |
| >10% | 0.50 | 0.31-0.81 | **0.005** |
| Neoadjuvant-targeted therapy |  |  |  |
| No | 1 |  |  |
| Yes | 0.35 | 0.17-0.73 | **0.005** |
| NAC cycles |  |  | 0.170 |
| ≤4 | 1 |  |  |
| 5-7 | 0.93 | 0.60-1.45 | 0.753 |
| ≥8 | 0.60 | 0.34-1.05 | 0.074 |
| ypTis/0N0 |  |  |  |
| No | 1 |  |  |
| Yes | 0.34 | 0.15-0.74 | **0.007** |

Abbreviations: TILs, tumor infiltrating lymphocytes; cT, clinical tumor stage; cN, clinical nodal stage; HER2, human epidermal growth factor receptor 2; HR, hormonal receptor; TNBC, triple negative breast cancer; NAC, neoadjuvant chemotherapy.

**Table S7. Univariate analysis of factors associated with OS in whole population.**

| Characteristics | Whole population |
| --- | --- |
| Age | 0.113 |
| Menopausal status | 0.052 |
| cT | **<0.001** |
| cN | 0.561 |
| Pathology | **0.022** |
| Grade | **0.015** |
| ER | **0.008** |
| PR | 0.079 |
| HER2 | **0.005** |
| Ki67 | 0.103 |
| Molecular subtypes | **<0.001** |
| Pre-NAC TILs | **0.016** |
| NAC regimens | **0.012** |
| Neoadjuvant-targeted therapy | **0.004** |
| NAC cycles | **0.048** |
| Breast surgery | **0.027** |
| Axillary surgery | 0.697 |
| ypTis/0N0 | **0.010** |

Abbreviations: TILs, tumor infiltrating lymphocytes; cT, clinical tumor stage; cN, clinical nodal stage; ER, estrogen receptor; PR, progesterone receptor; HER2, human epidermal growth factor receptor 2; NAC, neoadjuvant chemotherapy.

**Table S8. Multivariate analysis of factors associated with OS in whole population.**

| Characteristics | Multivariate | | |
| --- | --- | --- | --- |
|  | OR | 95%CI | p-value |
| cT |  |  | **<0.001** |
| 1 | 1 |  |  |
| 2 | 0.81 | 0.33-2.00 | 0.653 |
| 3 | 1.54 | 0.56-4.21 | 0.399 |
| 4 | 4.15 | 1.54-11.23 | **0.005** |
| Pathology |  |  |  |
| IDC | 1 |  |  |
| Others | 1.33 | 0.62-2.86 | **0.460** |
| ER |  |  |  |
| Negative | 1 |  |  |
| Positive | 0.33 | 0.20-0.56 | <0.001 |
| HER2 |  |  |  |
| Negative | 1 |  |  |
| Positive | 1.11 | 0.41-3.04 | 0.840 |
| Grade |  |  | **0.003** |
| I-II | 1 |  |  |
| III | 3.69 | 1.68-8.10 | **0.001** |
| NA | 3.27 | 1.52-7.02 | **0.002** |
| Molecular subtypes |  |  | **0.533** |
| HR+HER2- | 1 |  |  |
| HER2+ | 1.13 | 0.41-3.09 | **0.816** |
| TNBC | 1.74 | 0.61-4.95 | **0.301** |
| Pre-NAC TILs |  |  |  |
| ≤10% | 1 |  |  |
| >10% | 0.54 | 0.29-1.01 | **0.053** |
| Neoadjuvant-chemotherapy |  |  | **0.093** |
| Anthracycline | 1 |  |  |
| Taxanes | 3.05 | 1.07-8.65 | **0.036** |
| Anthracycline+taxanes | 1.90 | 0.74-4.88 | **0.184** |
| Neoadjuvant-targeted therapy |  |  |  |
| No | 1 |  |  |
| Yes | 0.22 | 0.09-0.53 | **0.001** |
| NAC cycles |  |  | 0.597 |
| ≤4 | 1 |  |  |
| 5-7 | 1.12 | 0.60-2.10 | 0.721 |
| ≥8 | 0.77 | 0.34-1.78 | 0.542 |
| Breast surgery |  |  |  |
| Mastectomy | 1 |  |  |
| BCS | 0.17 | 0.02-1.25 | 0.081 |
| ypTis/0N0 |  |  |  |
| No | 1 |  |  |
| Yes | 0.34 | 0.12-0.97 | **0.043** |

Abbreviations: TILs, tumor infiltrating lymphocytes; cT, clinical tumor stage; ER, estrogen receptor; HER2, human epidermal growth factor receptor 2; HR, hormonal receptor; TNBC, triple negative breast cancer; NAC, neoadjuvant chemotherapy; BCS, breast conservative surgery.
